# Comparison of bipolar hemiarthroplasty and total hip arthroplasty with dual mobility cup in the treatment of old active patients with displaced neck of femur fracture: A retrospective cohort study

**DOI:** 10.1016/j.amsu.2019.07.025

**Published:** 2019-07-13

**Authors:** Shah Fahad, Muhammed Zohaib Nawaz Khan, Talal Aqueel, Pervaiz Hashmi

**Affiliations:** Department of Surgery, AKUH, Pakistan

**Keywords:** Bipolar hemiarthroplasty, dual mobility cup, Dislocation, Mortality

## Abstract

**Background:**

The standard treatment of displaced femoral neck fracture is arthroplasty. THA is reportedly superior to BHA in terms of hip pain, function and reoperation rate. On the other hand THA has a higher rate of dislocation. Total hip replacement with dual mobility cup increases the range of motion and reduces the chances of dislocation. The aim of this study is to compare the functional outcome, rate of dislocation, complications and mortality between BHA and THA with dual mobility cuff for the treatment of displaced neck of femur fracture.

**Patients and method:**

This is a non-commercialized retrospective cohort study conducted at our tertiary care level 1 trauma centre. Patients of age group 60 years and above who underwent hip arthroplasty (BHA or THA with dual mobility implant) between 2015 and 2017 for displaced neck of femur fracture with a complete follow up for one year were included. Both groups were assessed for postoperative surgical complications including dislocation, fracture, surgical site infection, and medical complications, one-year mortality and functional outcome were analysed via Harris Hip Score (HHS) at the latest follow up.

**Results:**

Overall 104 patients were included in the study out of which 77 patients underwent BHA while 27 underwent THA with dual mobility cup. Baseline characteristics were found to be similar in both groups. Mean pre-op HHS for bipolar group was found to be 71.01 while for THA with dual mobility cup group it was 73.52 with the difference being statistically insignificant (P = 0.12). Mean post-op HHS for bipolar group was noted to be 68.82 whereas for THA with dual mobility cup group it was 76.81. The difference was found to be statistically significant with a P-value of <0.01. With regards to post-operative complications and one-year post-operative mortality, no significant difference was noted between both groups.

**Conclusion:**

In relatively young and active elderly patients with displaced neck of femur fracture, a THA with dual mobility cuff provides better hip functional outcome, does not increase mortality or morbidity as compared to BHA and can be considered as primary treatment modality.

## Highlights

• The standard treatment of displaced femoral neck fracture is arthroplasty.
• Total Hip Arthroplasty is reportedly superior to Bipolar Hemiarthroplasty in terms of hip pain, function and reoperation rate.
• Total Hip Arthroplasty has a higher rate of dislocation rate than Bipolar Hemiarthroplasty.
• Total Hip Arthroplasty with Dual mobility cup increases the functional outcome and reduces the chances of dislocation.

## 1 Background

The standard treatment of displaced femoral neck fracture is arthroplasty, as this treatment allows early mobilization of the patient [1]. Bipolar hemiarthroplasty (BHA) and total hip arthroplasty (THA) are widely accepted methods of hip replacement after fracture. THA is reportedly superior to BHA in terms of hip pain, function and reoperation rate. On the other hand THA has a higher rate of dislocation [2,3] while BHA is a less complicated surgery, has shorter operation time, less blood loss and lower initial cost [4].

Dual mobility implant concept was introduced by Prof. Gilles Bousquet. This dual mobility implant comprises of a prosthetic head which is mobile within a polyethylene liner and further the liner articulates with the acetabular metal cup that increases the range of motion, mobility and provides stable construct. This increases the functional outcome and reduces the chances of dislocation [5,6].

The aim of this study is to compare the functional outcome, rate of dislocation, complications and mortality between BHA and THA with dual mobility cuff for the treatment of displaced neck of femur fracture.

## 2 Materials and methods

### 2.1 Patients

A retrospective cohort study was conducted at our tertiary care level 1 trauma centre. ERC approval was taken from Ethical Review Committee of the hospital. Patients of age group 60 years and above who underwent hip arthroplasty (BHA or THA with dual mobility implant) between 2015 and 2017 for displaced neck of femur fracture with a complete follow up for one year were included. Patients who were excluded from the study included those who underwent hip arthroplasty for other indications, those younger than 60 years, with pathological fractures, missing records and those lost to follow up. All surgeries were performed by experienced orthopaedic surgeons with specialized training in arthroplasty. The work has been reported in line with the STROCSS criteria [7].

### 2.2 Surgery and post-operative care

All procedures were performed by fellowship trained consultants and as per surgeon preference through lateral or posterior approach. Standard 130 mm cementless porous coated femoral stems were used. A comparable population in terms of age, gender and functional status was included for both bipolar head and dual mobility cup. Dual mobility cups were made up of titanium alloy with hydroxyapatite coating stabilized by uncemented press fit technique with additional initial stability using two pegs and a single screw quadrant. Head was made up of standard stainless steel with high molecular polyethylene size measuring 28 or 32 mm as per cup size. The femoral stem and acetabular component was anteverted by 15° with an inclination of 40°.

Post operatively the patients ambulated using a walker for 2–4 weeks. Three doses of cephalosporin antibiotic therapy were given. Quadriceps muscle strengthening exercises were started from day one after surgery. Low molecular weight heparin as DVT prophylaxis was given to all patients. Patients were discharged on third post-operative day and followed in clinic after one week. After two weeks post-operatively stitches were removed.

The functional outcome was evaluated before the first stage operation and at the time of the latest follow-ups.

### 2.3 Outcome variables

Both groups were assessed for postoperative surgical complications including dislocation, peri-prosthetic fracture and surgical site infection, medical complications like renal failure, myocardial infarction, and pulmonary embolism. Also one-year mortality and functional outcome were analysed via Harris Hip Score (HHS) at the latest follow up.

### 2.4 Statistical analysis

Data was analysed using SPSS version 20. Chi-square test was used to compare categorical variables whereas independent sample T-test was used to compare continuous variables. P-value of <0.05 was considered significant.

## 3 Results

Overall 104 patients were included in the study out of which 77 patients underwent BHA while 27 underwent THA with dual mobility cup. Baseline characteristics were found to be similar in both groups as shown in Table 1. Mean pre-op HHS for bipolar group was found to be 71.01 while for THA with dual mobility cup group it was 73.52 with the difference being statistically insignificant (P = 0.12).

**Table 1:** Baseline Characteristics of study population.

Characteristics	Surgery type		P-value
	BHA group	THA with dual mobility cup group	
	N = 77	N = 27	
Age (years)	71.1 ± 10.9	69.3 ± 9.0	0.46
Gender			0.48
Male	31 (40.3)	13 (48.1)	
Female	46 (59.7)	14 (51.9)	
Co-morbidities			0.88
None	12 (15.6)	5 (18.5)	
One	24 (31.2)	10 (37.0)	
Two	19 (24.7)	5 (18.5)	
More than two	22 (28.6)	7 (25.9)	
Hospital stay (days)	7.2 ± 4.2	5.7 ± 3.3	0.10
Follow-up duration (months)	20.6 ± 6.6	19.0 ± 5.4	0.25
Pre-op HHS	71.0 ± 10.5	73.5 ± 5.4	0.12
Post op HHS	68.82 ± 10.4	76.81 ± 5.4	0.01

In terms of outcome variables, mean post-op HHS for bipolar group was noted to be 68.82 whereas for THA with dual mobility cup group it was 76.81. The difference was found to be statistically significant with a P-value of <0.01. With regards to post-operative complications, no significant difference was noted between both groups as shown in Table 2. The one-year mortality for bipolar group was found to be 5.7% compared to none in THA with dual mobility cup group, however the difference was insignificant (P = 0.23).

**Table 2:** Post-operative outcomes in study population.

Outcome measures	BHA group N = 77	THA with dual mobility cup group N = 27	P-value
Complications			0.55
None	66 (85.7)	23 (85.2)	
Pulmonary Embolism	1 (1.3)	1 (3.7)	
Myocardial Infarction	2 (2.6)	0 (0.0)	
Surgical site infection	2 (2.6)	1 (3.7)	
Acute Kidney Injury dislocation	2 (2.6)	2 (7.4)	
One-year Mortality	4 (5.2)	0 (0.0)	0.23

## 4 Discussion

Our study showed no statistically significant difference in mortality, morbidity between both groups, while THA with dual mobility cuff group has better functional outcome than BHA group in treatment of displaced neck of femur fracture in elderly patients.

THA is the preferred treatment modality for healthy elderly patient with displaced neck of femur fracture because of its better functional results and low revision rate [8], but this procedure is commonly complicated by high dislocation rate. Several studies preferred BHA in healthy elderly patient [9,10] because of low dislocation rate and good functional outcome but this procedure is complicated by high revision rate. Hence in active healthy elderly patients there is still controversy over whether total hip arthroplasty or bipolar hip arthroplasty should be selected for displaced neck of femur fracture [11,12].

Dual mobility total hip reduces the risk of dislocation, cause less impingement, decreases friction and wear [13]. It also increases range of motion [14]. The concept of dual mobility cuff was introduced by Bousquet in the late 1970s. This construct combines the concepts of low friction arthroplasty with large head articulation. The intervening polyethylene liner between the prosthetic head and outer metal shell provides two bearings, where motion preferentially occurs at the inner bearing and the outer bearing is engaged at the extremes of motion. This increases effective head size and improves head-to- neck ratio. Thus it improves range of motion, reduces impingement and provides stability [5,6].

Mortality in elderly patients with hip fracture is a major concern. Several studies assessed the one-year mortality rate after BHA as compared to THA and they showed no significant difference between them [4,15,16]. In our study we found no significant difference in one-year mortality between the two groups.

Dislocation after hip arthroplasty is a key issue. Compared to bipolar hemiarthroplasty, total hip replacement is associated with high dislocation rate [[17], [18], [19], [20]]. Dual mobility THA is associated with reduced risk of dislocation without increasing the mortality [21].

The principle concerns on THA dual mobility are intra-prosthetic dislocations and increased wear at the liner. Intra-prosthetic dislocation occurs from dislodgment of prosthetic head from the mobile liner. This is a long term complication and results from wear at the retentive rim of the polyethylene liner. With the improvement of implant design the incidence of this complication is dropped to 0% at 6 years and 0.28% at 10 years follow up. In our study we didn't encounter intra-prosthesis dislocation at mean follow up of 19.0 ± 5.4 month.

There was high revision surgery rate with BHA group compared to THA group in the literature. The high revision is related to acetabular erosion [12]. In our study we did not encounter revision surgery. One reason for this is short follow up.

Limitations of our study include retrospective study design and short follow up.

We recommend prospective randomize trial with large sample size.

Compared to hemiarthroplasty total hip replacement causes marked improvement in functional status [11,22]. Many studies showed better outcomes after THA.

## 5 Conclusion

In relatively young and active elderly patients with displaced neck of femur fracture, a THA with dual mobility cuff provides better hip functional outcome, less dislocation rate and does not increase mortality or morbidity as compared to BHA and can be considered as primary treatment modality **i**n the treatment of old active patients with displaced neck of femur fracture.

## Ethical approval

Hospital ethical review committee approval was taken.

## Source of funding

None.

## Author contribution

Shah Fahad: first proposal, data collection, analysis and manuscript writing and editing.

Muhammag Zohaib Nawaz Khan: data analysis and manuscript writing and editing.

Talal Aqueel: review and editing.

Pervaiz Hashmi: review and editing.

## Conflicts of interest

None.

## Registration of research studies

Registration of the study in data registry with research registry NCT03875014.

## Guarantor

Pervaiz Hashmi.

Shah Fahad.

## Consent

Not applicable.

## Provenance and peer review

Not commissioned, internally reviewed.
